# Supplementary material for: Redefining transcriptional regulation of the APOE gene and its association with Alzheimer’s disease
Source: PLoS One. 2020 Jan 24;15(1):e0227667. doi: 10.1371/journal.pone.0227667 (PMC6980611; doi:10.1371/journal.pone.0227667)
Supplement: S1 Fig — (A) Partial sequence of APOE Ex 3 (blue font) and 4 (black font) shows locations of regular splice site, back-splice sites that contain a common AGCTGC sequence (highlighted in yellow), and the two outward primers (orange arrows) that amplify APOE circRNAs. Note that both circRNAs contain the ε4-determing SNP (rs429358, red font with underline). (B) Stem-loop formation of L-circRNA from original template was predicted using the RNAstructure program (https://rna.urmc.rochester.edu/RNAstructureWeb) with a probability of >75%. (C) The same analysis predicted a probability of >95% for the stem-loop formation of S-circRNA. circRNA: circular RNA; Ex: exon; L: large; S: small; SNP: single-nucleotide polymorphism. (PDF) [file pone.0227667.s001.pdf]

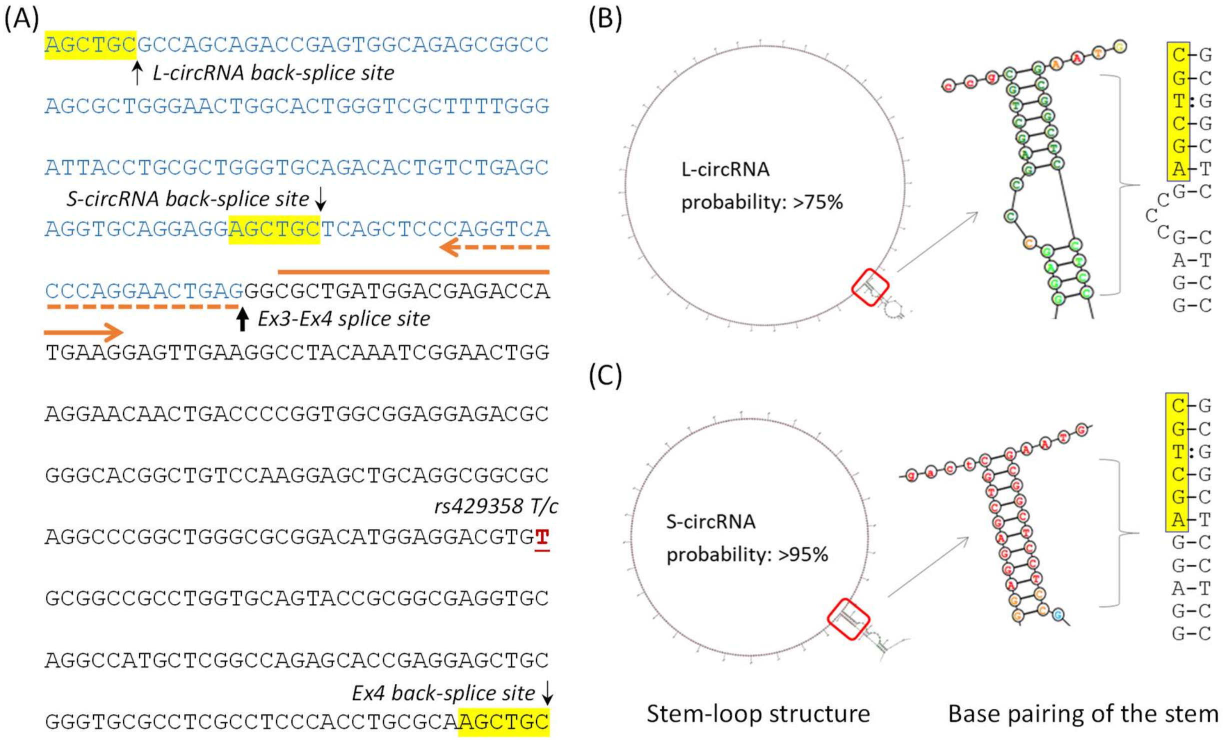

(A)
AGCTGCGCCAGCAGACCGAGTGGCAGAGCGGCC
L-circRNA back-splice site
AGCGCTGGGAACTGGCACTGGGTCGCTTTTGGG
ATTACCTGCGCTGGGTGCAGACACTGTCTGAGC
S-circRNA back-splice site
AGGTGCAGGAGGAGCTGCTCAGCTCCCAGGTCA
CCCAGGAACTGAGGGCGCTGATGGACGAGACCA
Ex3-Ex4 splice site
TGAAGGAGTTGAAGGCCTACAAATCGGAACTGG
AGGAACAACTGACCCCGGTGGCGGAGGAGACGC
GGGCACGGCTGTCCAAGGAGCTGCAGGCGGCGC
rs429358 T/c
AGGCCCGGCTGGGCGCGGACATGGAGGACGTGT
GCGGCCGCCTGGTGCAGTACCGCGGCGAGGTGC
AGGCCATGCTCGGCCAGAGCACCGAGGAGCTGC
Ex4 back-splice site
GGGTGCGCCTCGCCTCCCACCTGCGCAAGCTGC
(B)
L-circRNA
probability: >75%
C-G
G-C
T:G
C-G
G-C
A-T
G-C
C
C
C
G-C
A-T
G-C
G-C
(C)
S-circRNA
probability: >95%
C-G
G-C
T:G
C-G
G-C
A-T
G-C
G-C
A-T
G-C
G-C
Stem-loop structure
Base pairing of the stem
