# Supplementary figures and images for: Redefining transcriptional regulation of the APOE gene and its association with Alzheimer’s disease
Source: PLoS One. 2020 Jan 24;15(1):e0227667. doi: 10.1371/journal.pone.0227667 (PMC6980611; doi:10.1371/journal.pone.0227667)

## Frontal Lobe

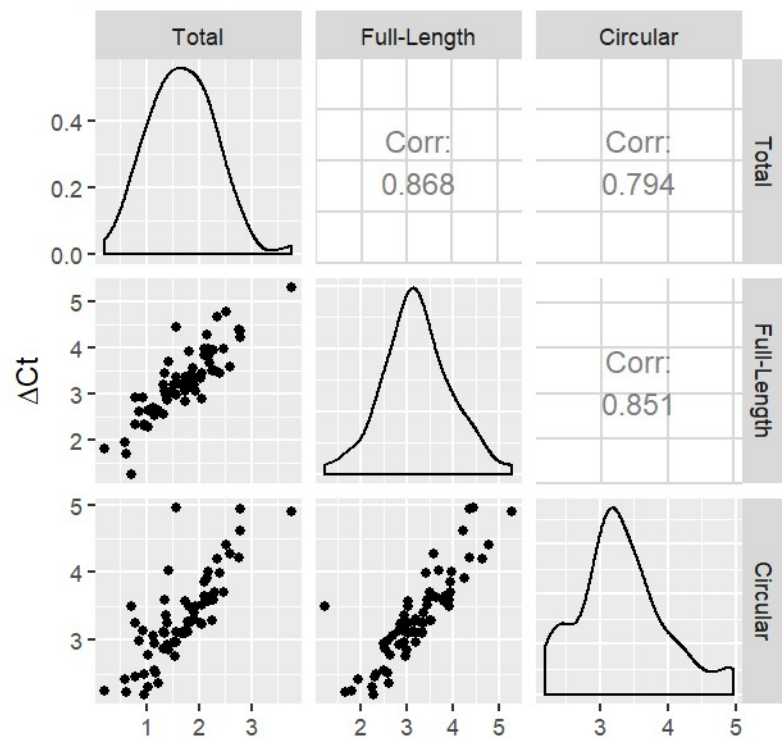

## Cerebellum

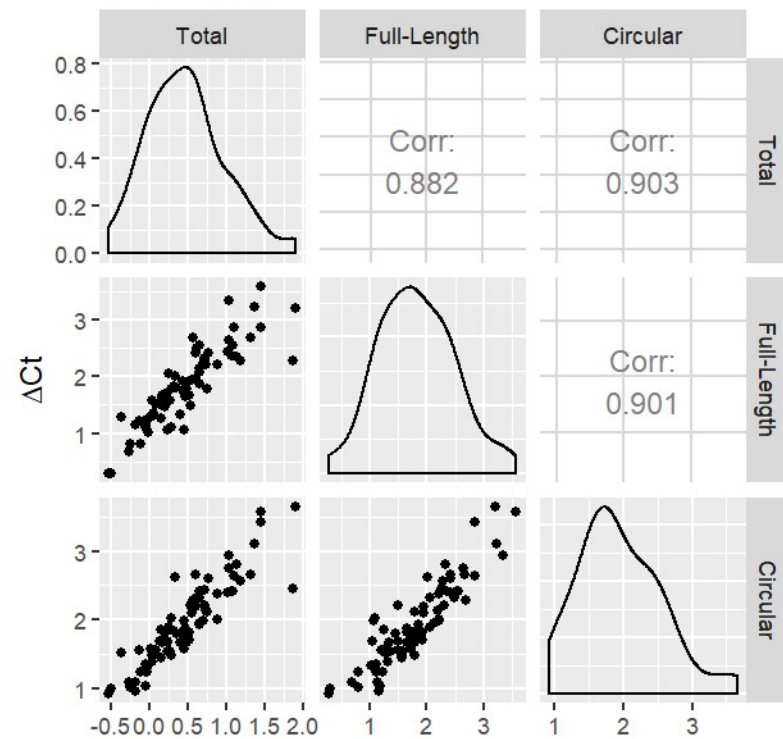

Supplement: S2 Fig — Expression levels of three APOE RNAs (circular, full-length, and total) are plotted as values of ΔCt (Ct of APOE RNA–Ct of ACTB RNA) in both frontal lobe (left panel) and cerebellum (right panel). Plots on the diagonal are the empirical density of expression levels for that particular RNA type. Corr: correlation; Ct: cycle threshold. (PDF) [file pone.0227667.s002.pdf]

(A)

Combined Samples  
 $r = 0.1$  ;  $p = 0.197$

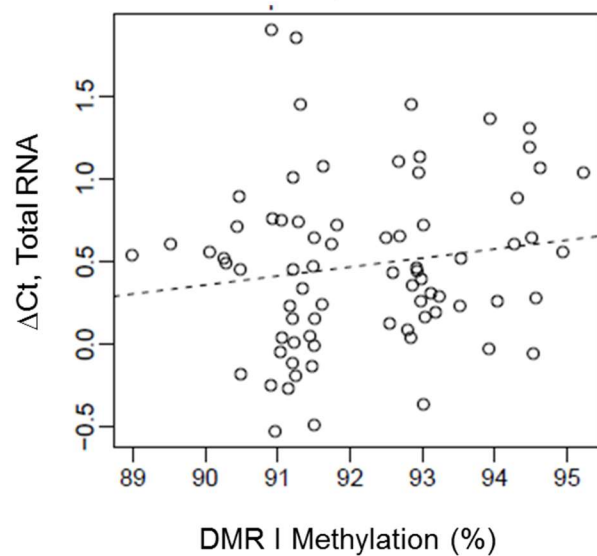

(B)

Control (Blue):  $r = -0.11$  ;  $p = 0.85$   
AD (Red):  $r = 0.21$  ;  $p = 0.10$

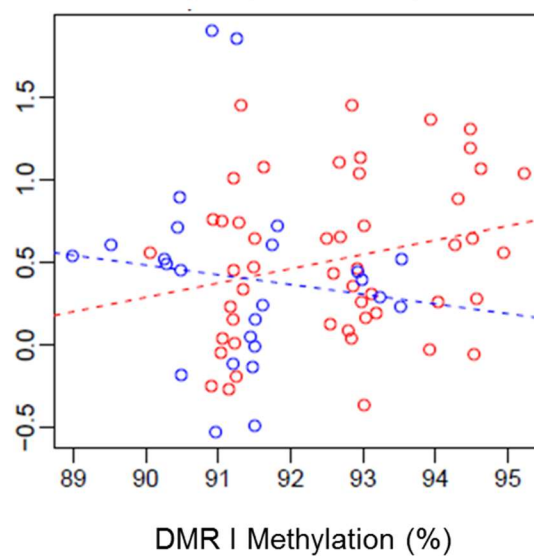

Supplement: S3 Fig — Total APOE RNA ΔCt is plotted against mean methylation across DMR I (CpG site #11–37). (A) All cerebellum samples including both AD and control subjects. (B) Plot separating AD (red) from control (blue). Dashed lines and p-values are associated with the respective fitted linear regression models. Note that lower ΔCt values represent higher expression levels. AD: Alzheimer’s disease; Ct: cycle threshold; DMR I: differentially methylated region 1. (PDF) [file pone.0227667.s003.pdf]

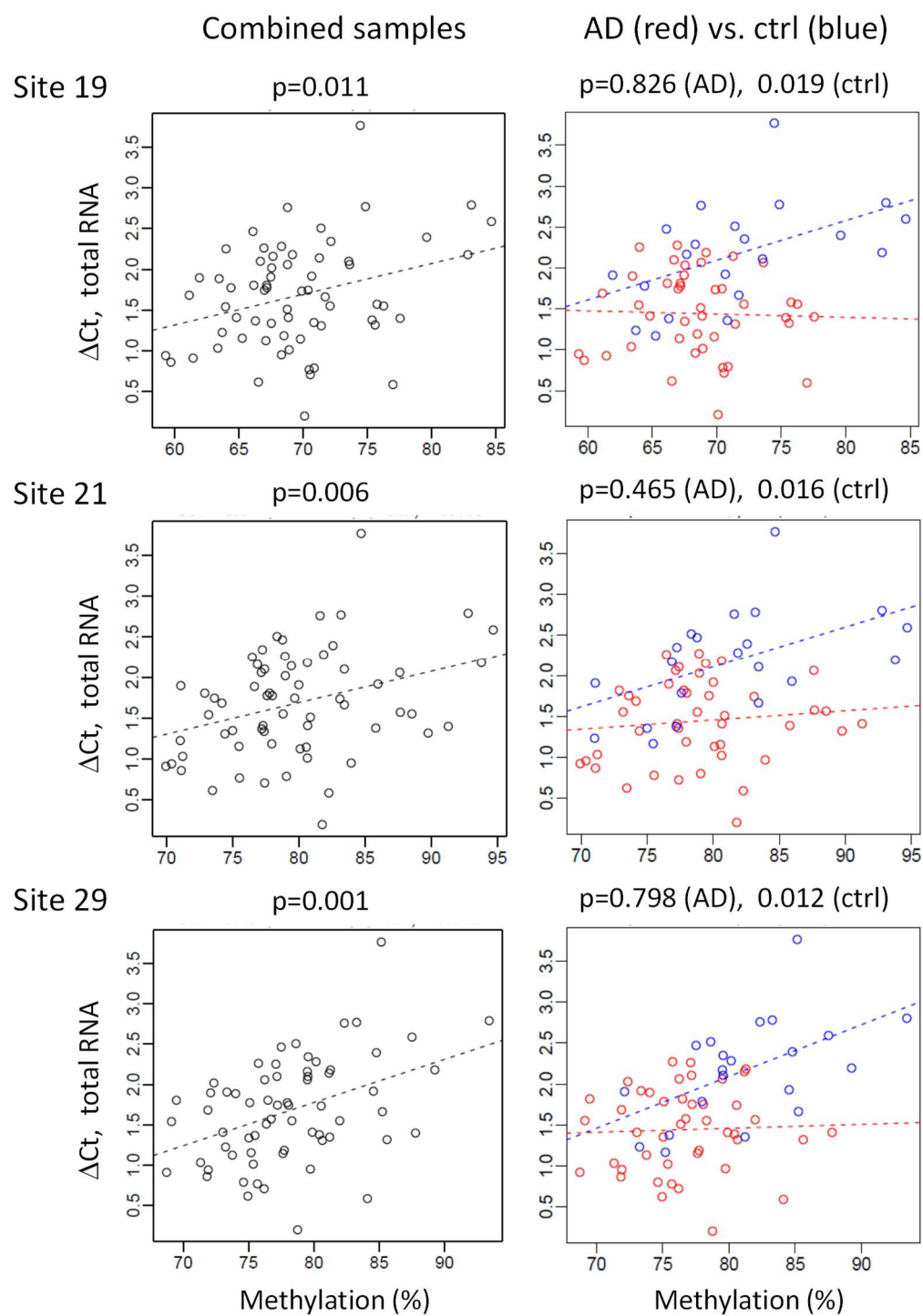

Supplement: S4 Fig — Total APOE RNA ΔCt is plotted against DNA methylation levels of individual APOE CpG sites (#19, 21, and 29) for all frontal lobe samples (includes both AD and control samples; left panel) and separated AD (red) and control (blue) samples (right panel) with respective linear fit lines (dashed) and uncorrected correlation p-values. Note that lower ΔCt values represent higher expression levels. AD: Alzheimer’s disease; Ct: cycle threshold; Ctrl: control. (PDF) [file pone.0227667.s004.pdf]

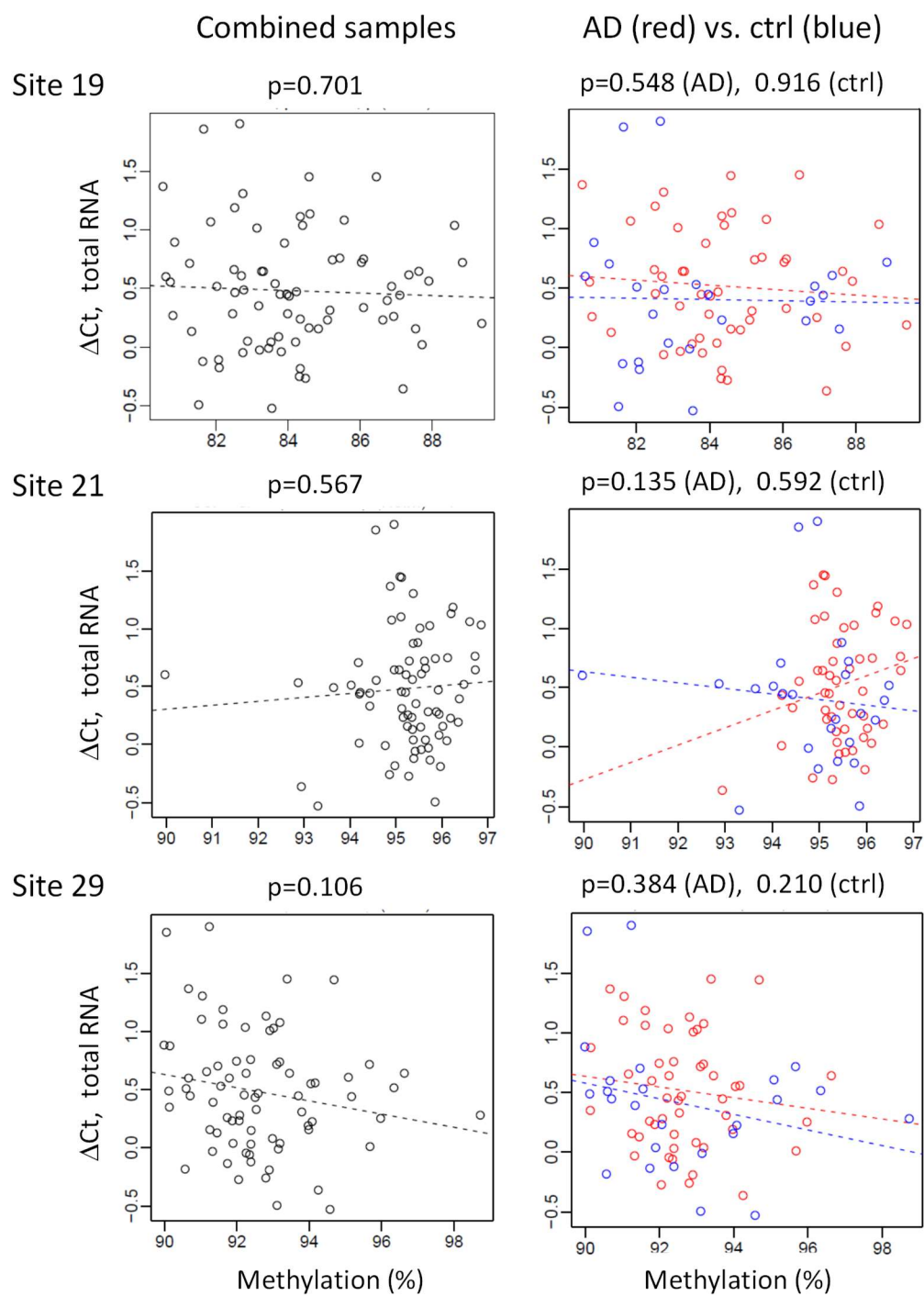

Supplement: S5 Fig — Total APOE RNA ΔCt is plotted against DNA methylation levels of individual APOE CpG sites (#19, 21, and 29) for all cerebellum samples (includes both AD and control samples; left panel) and separated AD (red) and control (blue) samples (right panel) with respective linear fit lines (dashed) and uncorrected correlation p-values. Note that lower ΔCt values represent higher expression levels. AD: Alzheimer’s disease; Ct: cycle threshold; Ctrl: control. (PDF) [file pone.0227667.s005.pdf]
