# Supplementary material for: Redefining transcriptional regulation of the APOE gene and its association with Alzheimer’s disease
Source: PLoS One. 2020 Jan 24;15(1):e0227667. doi: 10.1371/journal.pone.0227667 (PMC6980611; doi:10.1371/journal.pone.0227667)
Supplement: S1 Table — (PDF) [file pone.0227667.s006.pdf]

Target	Primer	Sequence 5'-	Comment
***APOE* circRNA**			
Conventional PCR			
	AE_circRNA_F1	CGCTGATGGACGAGACCATGAAG	Sense primer for both L- and S-
	AE_circRNA_R1	CTCAGTTCCTGGGTGACCTG	Antisense primer for both L- and S-
TaqMan assay			
	AE_circRNA_F3	TGGTGCAGTACCGCGGCGAG	Sense primer for both L- and S-
	AE_circRNA_R3	CTGGGAGCTGAGCAGCTTGCGC	Antisense primer for S- only
	AE_circRNA_R4	TGCCACTCGGTCTGCTGGCG	Antisense primer for L-only
	AE_circRNA_Probe	TCGGCCAGAGCACCGAGGAG (5' 6-FAM/ZEN/3' IBFQ)	for both L- and S-; ordered from Integrated DNA Technologies
***APOE* full-length mRNA** (TaqMan assay)			
	AE_Full-RNA_F1	CCTCAAGAGCTGGTTCGAG	Sense primer
	AE_Full-RNA_R1	TCGGCGTTCAGTGATTGTC	Antisense primer
	AE_Full-RNA_ Probe	TGGTGGAGAAGGTGCA (5' FAM/3' MGB)	Ordered from Thermo Fisher
***APOE* total RNA** (TaqMan assay)			Thermo Fisher (Hs00171168_m1)
***ACTB* mRNA** (TaqMan assay)			Thermo Fisher (Hs01060665_g1)

ACTB: β Actin; circRNA: circular RNA; L: large; S: small.
