# Supplementary material for: Redefining transcriptional regulation of the APOE gene and its association with Alzheimer’s disease
Source: PLoS One. 2020 Jan 24;15(1):e0227667. doi: 10.1371/journal.pone.0227667 (PMC6980611; doi:10.1371/journal.pone.0227667)
Supplement: S2 Table — (PDF) [file pone.0227667.s007.pdf]

RNA type	Frontal lobe		Cerebellum	
	AD	Control	AD	Control
Circular	0.33 (0.08)	0.33 (0.07)	0.36 (0.06)	0.38 (0.08)
Full-length	0.35 (0.09)	0.35 (0.08)	0.37 (0.07)	0.48 (0.11)
Truncated	0.32 (0.14)	0.31 (0.13)	0.27 (0.11)	0.16 (0.16)

Note: Fractions, reported as mean (SD), represent FC between expression level for total versus circular, full-length, or truncated RNA. For circular and full-length RNA, fractions were computed as FC (target) = $2^{-\Delta\Delta Ct}$, where $\Delta\Delta Ct = \Delta Ct$ (target) – ΔCt (total). For truncated mRNA, fractions were computed as FC (truncated) = 1 – FC (circular) – FC (full-length). AD: Alzheimer's disease; Ct: cycle threshold; FC: fold change; SD: standard deviation.
