# Supplementary material for: Redefining transcriptional regulation of the APOE gene and its association with Alzheimer’s disease
Source: PLoS One. 2020 Jan 24;15(1):e0227667. doi: 10.1371/journal.pone.0227667 (PMC6980611; doi:10.1371/journal.pone.0227667)
Supplement: Attachment — Submitted filename: Rebuttal Letter_191213.docx [file pone.0227667.s008.docx]

**AUTHOR RESPONSE TO REVIEWERS’ COMMENTS**

We thank the Reviewers for their effort thoroughly reviewing this manuscript. Their insightful comments have led us to make many improvements to this revision. Overall, the Reviewers evaluated the manuscript positively, but they also raised some concerns. To address each reviewer’s specific concerns, we have generated a point by point discussion outlined below:

**Reviewer 1**

1. It was very interesting that the authors identified two novel circRNAs (termed as S-circRNA and L-circRNA) using a genomic walking strategy in conjunction with RT-PCR. As we all known, there are other non-coding RNAs besides circRNAs, like microRNAs and lncRNAs. So, are there any reports about the microRNAs or lncRNAs of APOE? Or have the authors ever done any related tests of microRNAs or lncRNAs of APOE although the authors shortly addressed the question in discussion?

[Response] We did not design comprehensive experiments to search for other non-coding RNA of *APOE*; however, there is evidence of their existence. A natural antisense *APOE* RNA transcript has been reported in the study of Seitz et al. (Ref# 61), and a long non-coding *APOE* RNA (lnc-ZNF296-6) has been defined in a lncRNA database (LNCipdia). To the best of our knowledge, no known *APOE* microRNA has been identified. We queried multiple miRNA databases (miRbase, MiRDB, TarBase) and did not find any record of microRNA originating from the *APOE* gene. This information has been integrated into the revision (marked-up version: lines 578-583, unmarked version: lines 564-569).

2. The authors used the human cell lines, including HepG2 (hepatocellular carcinoma cells), SH-SY5Y (neuroblastoma cells), and U87 (glioma 164 cells) to quantified APOE RNAs. The rationale to choose these three cell lines should be clarified more directly in text.

[Response] We thank the Reviewer for bringing our attention to this shortcoming. Our study is focused on *APOE* RNA expression. Human liver cells and glial cells of the CNS are the two major cell types that express *APOE*. Thus, we selected our cell models to best mimic these cell-types including hepatocyte (HepG2) and glia (U87 and LN-229) cells. We also included a neuronal related cell (SH-SY5Y) for comparison purposes. This information has been integrated into the revision (marked-up version: lines 298-301, unmarked version: lines 286-289).

3. In line 171, “we used antibodies specific to either the N-terminus or the C-terminus of the APOE protein and performed semiquantitative Western blot analysis on lysates from HepG2 cells to inspect whether the truncated mRNAs can be translated into proteins.” The results in SH-SY5Y and U87 cell lines should be added in the article

[Response] In response to this comment, we have run new Western blots on cell lysates from all three cell lines, including HepG2, SH-SY5Y and U87. However, the ApoE expression in SH-SY5Y and U87 cells was too low to quantify using our standard curve method. Our ELISA data aligns with this finding in that ApoE secretion from SH-SY5Y cells was over 200 times less than that from HepG2. From this we have concluded that semiquantitative Western blot is not an appropriate method to measure ApoE protein with low expression level of ApoE. This information has been integrated into the revision (marked-up version: lines 315-317, unmarked version: lines 302-304).

4. In line 304, the usage of 5-Aza-dCTP for the inhibition of DNA methylation should be addressed clearly.

[Response] The chemical 5-aza-dC is a suicidal analog of cytosine, acting as a DNA methyltransferase (DNMT) inhibitor (Ref. # 58). DNMT transfers a methyl group to the C5 position of Cytosine at CpG dinucleotides. When 5-aza-dC is incorporated into DNA, a covalent bond is formed between 5-aza-dC and DNMT. Because C5 position of cytosine is replaced with nitrogen in 5-aza-dC, the methyl transfer reaction cannot take place and the DNMT is irreversibly bound to the DNA. The treatment of 5-aza-dC results in a depleted pool of DNMT, leading to a decrease in DNA methylation. This information has been integrated into the revision (marked-up version: lines 469-475, unmarked version: lines 456-462).

5. In lines 308-310 and figure 6, the western blot of ApoE protein should be also used to demonstrate the effects of 5-Aza-dCTP treatment.

[Response] We have shown that the Western blot is not an appropriate method to quantify low levels of ApoE protein expressed in cells. Also, the majority of ApoE protein is secreted with only a small portion retained in the cells. To best address this concern, instead of using Western blot, we performed a new ELISA experiment to quantify secreted ApoE levels in culture media from 5-aza-dC treated and un-treated cells. The results are shown in (Fig 6A) of the revision (marked-up version: lines 469-485, unmarked version: lines 466-472).

6. In line 317, “We performed ChIP on HepG2 cells using an anti-MECP2 antibody, and we then PCR quantified ChIP-recovered DNA fragments of three APOE gene regions.” The results in SH-SY5Y and U87 cell lines should be also added and discussed in the article.

[Response] We have performed a new ChIP experiment to address this concern. Given that U87 produces very low levels of *APOE* RNA, we have instead used another glioblastoma cell line LN-229 which has higher *APOE* RNA expression than U87. Results of ChIP-qPCR on LN-229 and SH-SY5Y have been integrated into (Fig 6B) of the revision. This new result aligns with our previous 5-aza-dC experiment shown in Fig. 6A. (marked-up version: lines 498-505, unmarked version: lines 485-491).

7. Women have higher risk to develop AD than men. Epidemiological studies revealed that two-thirds of AD patients are women. In line 259, “When APOE ε4 status and sex were included as covariates, effect sizes increased slightly for all three RNAs, though the increases were more pronounced for circRNA (from 1.21 to 1.45) and 260 total RNA (from 1.32 to 1.43) than for full-length mRNA (from 1.04 to 1.05).” Can the authors make some explanations about the correlation between the gender difference and APOE RNAs expression level differences?

[Response] We observed a slight increase in effect sizes for *APOE* RNAs when both gender and ε4 status were used as covariant. However, this change did not reach the level of statistical significance. While this subtle change could be a true biological effect, we cannot rule out the possibility that is an artifact of our small sample size. Given this uncertainty, we would like to avoid stretching our interpretation of this result, however, reporting it could stimulate more comprehensive follow up studies and generate more conclusive evidence on gender difference. We have clarified this further our revision (marked-up version: lines 419-423, unmarked version: lines 406-410).

8. The authors selected frontal lobe and cerebellum for this experiment because the frontal lobe is heavily affected by AD pathology whereas the cerebellum is minimally affected. As we all known, hippocampus plays a key role in learning and memory, which defected in AD patients. So, have the authors ever detected the expression of APOE RNAs in hippocampus? Or are there any reports in hippocampus?

[Response] Hippocampus is the most demanded brain tissue for AD research. Due to this demand and its small volume, hippocampal tissue is not available for a good portion of postmortem brain samples in our brain bank. Therefore, we selected frontal lobe, a tissue also heavily affected in AD, to conduct this study. Which allowed us to collect adequate number of brain tissues for these experiments.

9. Most of the references were outdated. So updated references should be cited.

[Response] In the revised manuscript, we have updated several of our cited references, which include reference #: 1, 2, 10, 22, 23, 33, 37,60, and 67.

10. Lack of references in several parts (for example, line 326-331).

[Response] We have added new references accordingly, which include references # 31-38.

**Reviewer 2**

Major concerns:

1. The authors state in the Author summary, Intro & Discussion that they assess how the association between APOE transcription and methylation influences the risk of AD, but this specific analysis is not performed and should be reworded.

[Response] We agree with the Reviewer’s comment that “influence the risk of AD” statement is not adequate. We have reworded this statement into “correlated the levels of RNA and DNA methylation with the risk of AD”. This change has been integrated in in both Introduction and discussion sections (marked-up version: lines 120 and 532, unmarked version: lines 111 and 518).

2. Results (lines 169-175): the authors conclude that since they did not observe truncated ApoE protein, that only full-length APOE mRNA can be translated into functional ApoE protein. This conclusion is only derived from the analysis of HepG2 cells – was this analysis similarly performed in SH-SY5Y and U87 cells and/or human samples since it is possible that truncated ApoE protein may be expressed in other cell lines or sample types? (and especially since the full-length mRNA constituted lower proportions of total RNA for SH-SY5Y & U87 cells)

[Response] We have run new Western blots on cell lysates from all three cell lines, including HepG2, SH-SY5Y and U87. However, the ApoE expression in SH-SY5Y and U87 cells was too low to quantify using our standard curve method. Our ELISA data aligns with this finding in that ApoE secretion from SH-SY5Y cells was over 200 times less than that from HepG2. From this we have concluded that semiquantitative Western blot is not an appropriate method to measure ApoE protein that lowly express ApoE. This information has been integrated into the revision (marked-up version: lines 315-317, unmarked version: lines 302-304).

3. Results (lines 169-175): for the Western blot assessments, does the C-terminus antibody target all 3 isoforms?

[Response] The ApoE2, E3, E4 protein isoforms related amino acid alterations occur at the protein’s N-terminus domain and do not alter the C-terminus domain. Therefore, the C-terminus antibody can target all three isoforms of ApoE protein with equal efficiency. We have addressed this point (marked-up version: lines 309-312, unmarked version: lines 296-299).

4. Results (lines 190-191): was there evidence of APOE circRNAs in all PMB samples since only some are shown in Fig3?

[Response] The *APOE* circRNAs were present in all human PMB samples tested. Using the *APOE* circRNA-specific TaqMan assay, we have detected their presence in all PMB samples isolated from cerebellum and frontal lobe. That result is shown in Figure 4. The gel electrophoresis picture on Figure 3 only show four representative samples as examples of RT-PCR amplified circRNA. We have added a statement to clarify this point (marked-up version: line 383, unmarked version: line 370).

5. Results (line 198): if the e4-determining SNP is present in both circRNAs, that seems to suggest that only e4 transcripts could be transcriptionally regulated by the circRNAs. Is this correct? This would be relevant for the results described in lines 258-262.

[Response] The ε4-determining SNP (rs429358) contains two alleles with one allele (C nucleotide) defining ε4 and another allele (T nucleotide) defining either ε2 or ε3. Therefore, *APOE* circRNAs can only be defined as either ε4 or ε2/ε3 containing circRNAs. Our expressional analysis using *APOE* genotype as covariates is only to differentiate ε4+ from ε4- samples. Thus, in the current design of our study, we cannot deduce *APOE* circRNAs to be associated with transcriptional regulation of specific ε2/ε3/ε4 alleles. Also, we did not design an experiment to specifically test how *APOE* genotype affects circRNA expression, but we did observe a slight increase in effect sizes for *APOE* RNAs when both gender and ε4 status were used as covariant. However, this change does not reach the level of statistical significance.

6. Results (lines 301-311): was this analysis performed on any PMB samples?

[Response] This analysis was not performed on PMB sample. Because incorporation of 5-aza-dC into DNA occurs during DNA replication; therefore, this procedure using 5-aza-dC treatment can only be performed on living cells with active DNA replication.

7. Discussion (lines 328-329): pathogenic effects are not just associated with quality or quantity, but more importantly, functional changes, which may not necessarily be associated quality or quantity.

[Response] We have revised this statement as follows to clarify our points. “Such an effect is usually carried out by the gene’s products including RNA and protein and can be explained by a change in qualities (structure or function) and quantity (expression level) that leads to physiological changes” (marked-up version: lines 525-527, unmarked version: lines 511-513).

8. Please provide methods text on the methylation analyses, including the sites assessed for the DMR 1 analyses and the genomic walking analyses. Please also clarify the number of replicates (biological & technical) used for each analysis.

[Response] We have added methods text as follows in the revision.

Quantification of DNA methylation levels by pyrosequencing was performed. Briefly, genomic DNA (500 ng each) was bisulfite converted using the EpiTect Bisulfite Kit (Qiagen). To evaluate the methylation status of the *APOE*’s DMR 1, we designed pyrosequencing assays to cover 27 CpG sites (CpG 11-37). PCR was performed on approximately 200 ng of bisulfite-converted DNA using PyroMark PCR kits (Qiagen) on a GeneAmp PCR System 9700 (Applied Biosystems, Grand Island, NY). Pyrosequencing was carried out on a PyroMark Q24 system (Qiagen) and data was analyzed using PyroMark Q24 software, version 2.0.6 (Qiagen). Bisulfite treatment controls were integrated as a quality control measure (marked-up version: lines 219-227, unmarked version: 207-214).

Genome Walking analysis was carried out by the following procedures. RT reactions were performed using a set of evenly spaced primers to prime either sense and antisense transcript of *APOE* exon 4. These cDNAs were subsequently amplified by PCR reactions using a series of spaced upstream primers that were designed to pair with each RT primer. All of the cDNA products were fully characterized by gel purification and DNA sequencing” (marked-up version: lines 201-207, unmarked version: lines 190-195).

Number of replicates of analyses: 1) ELISA three biological replicates and two technical replicates were assayed, 2) RT-qPCR: already written in Method: 2 independent RT reactions for cDNA prep, triplicates in qPCR assay using each cDNA prep, 3) Genome walking: PCR reactions using a series of RT primers and PCR primers were carried out 3 times; 4) 5-aza-dC: RT-qPCR and ChIP-qPCR assays were performed 3 times using cells independently treated with 5-aza-dC. This information has been added to their corresponding method sections.

Minor concerns:

1. Something to consider is that the cellular content of analyzed samples is a key factor as well for the methylation and expression analyses since APOE is primarily expressed in astrocytes and microglia.

[Response] We appreciate this Reviewer’s suggestion. In fact, we have published a work using flowcytometry to separate glial and neuron nuclei from a subset of postmortem brain samples that were used in this study (Foraker J et al. J Alzheimers Dis. 2015;48(3):745-55). Our finding suggested that the differential DNA methylation of *APOE* in AD mainly occurs in glial cell but not neurons.

2. Fig1: what transcripts are associated with the e2, e3, and e4 isoforms?

[Response] All RNA transcripts in Figure 1 contain both SNPs (rs429358 and rs7412) that determine the ε2/ε3/ε4 alleles, and we have not designed experiments to address the allelic expression of *APOE*. Therefore, currently, we do not know the relationship between *APOE* genotype and expression of specific *APOE* transcript variants. This will be a critical point to be addressed for the future studies.

3. As there are public RNAseq data on AD brains, it would be interesting to see if the identified circRNAs are present in those datasets.

[Response] We have discussed this point in our discussion (marked-up version: lines 584-585, unmarked version: lines 570-571).

4. Please provide statistics behind the analysis of reduced methylation & increased mRNA production (lines 301-311).

[Response]

For this analysis, bisulfite pyrosequencing and qRT-PCR assays were performed using three independently 5-aza-dC treated cells. Each sample’s qPCR measurement was performed in triplicate. Average and standard deviation of these multiple assays are calculated and fold change of levels of 5-aza-dC treated cells compared to those of untreated cells (set at 1.0 for untreated) are used for plotting in Figure 6A. We have integrated this information in the revision (marked-up version: lines 482-485, unmarked version: lines 469-472).

5. There are a few grammatical errors throughout.

[Response] We have tried our best to minimize the grammatical errors. A co-author (Andrew Shutes-David), who has a bachelor’s degree in English literature, has proofed read this manuscript.
